# Enduring the unseen burden: a qualitative analysis on long-term emotional impact of COVID-19 on long-term care workers

**DOI:** 10.1192/j.eurpsy.2024.1064

**Published:** 2024-08-27

**Authors:** L. Gonzalez-Spinoglio, A. Monistrol-Mula, C. Vindrola-Padros, S. Aguilar-Ortiz, B. Carreras, J. M. Haro, M. Felez-Nobrega

**Affiliations:** ^1^Research and Development Unit, Parc Sanitari Sant Joan de Déu, Institut Sant Joan de Déu, Sant Boi de Llobregat, Spain; ^2^Department of Targeted Intervention, University College London, London, United Kingdom; ^3^Department of Psychiatry and Psychology, Institute of Neuroscience, Hospital Clínic, Barcelona, Spain

## Abstract

**Introduction:**

Long-term care facilities, such as nursing homes and other assisted living facilities, have been hit particularly hard by the COVID-19. The overall pandemic created an enormous pressure on long-term care workers (LTCWs), making them particularly vulnerable to mental disorders. However, most of the existing evidence regarding the well-being of care professionals has predominantly focused on frontline healthcare workers.

**Objectives:**

This study aimed to identify long-term psychological needs of LTCWs derived from the COVID-19 pandemic, as part of a project that is developing an intervention to reduce psychological distress in this population group.

**Methods:**

We performed a qualitative study with a rapid research approach. Participants were recruited from long-term care facilities located in Catalonia, Spain. Between April and September 2022, we conducted semi-structured interviews inquiring about the most psychologically challenging stages of the pandemic, perceived emotions during those stages, main determinants of those emotions, and their emotional state at the time of the interview. We used a qualitative content analysis method with an inductive-deductive approach.

**Results:**

Thirty LTCWs participated in the study. Mean age was 44 (SD=11,4), 87% were females and one third were from foreign nationalities. The period of the pandemic with highest mental health burden was the outbreak, with almost every worker having experienced some form of emotional distress. Emotional distress persisted over time in more than half of participants, with fatigue and nervousness being the main emotions expressed at the time of the interview. High workload, feeling that pandemic times are not over and poor working conditions that have remained since then, have been the most frequently expressed determinants of such emotions.

**Conclusions:**

Long after the pandemic outbreak, emotional distress is still relevant. The persistent burden of psychological distress points to a need for institutions to take action to improve working conditions and promote employees’ wellbeing.

**Disclosure of Interest:**

None Declared

